# Impact of preoperative frailty and sarcopenia on short-term functional recovery after cardiac surgery in elderly patients: a prospective cohort study

**DOI:** 10.1016/j.jnha.2026.101003

**Published:** 2026-10-09

**Authors:** Guanglei Fan, Yang Zhang, Guoqiang Chen, Wanzhou Wang, Wenjing Zhao, Xiaowen Meng, Fuhai Ji

**Affiliations:** a Department of Anesthesiology, The First Affiliated Hospital of Soochow University, Suzhou, Jiangsu, People's Republic of China; b Institute of Anesthesiology, Soochow University, Suzhou, Jiangsu, People's Republic of China; c Department of Intensive Care Medicine, Affiliated Hospital of Xuzhou Medical University, 99 Huaihai West Road, Xuzhou City, 221006, People's Republic of China

**Keywords:** Sarcopenia, Frailty, Disability, Functional recovery, Cardiac surgery

## Abstract

**Background:**

Preoperative sarcopenia and frailty are associated with poor outcomes in elderly patients undergoing cardiac surgery; this study aimed to investigate the effects of sarcopenia and frailty on postoperative recovery in elderly patients.

**Methods:**

This prospective cohort study included 566 patients aged ≥60 years with an American Society of Anaesthesiologists (ASA) II–IV status who were scheduled for elective open-heart surgery between June 30, 2024, and April 5, 2026. Frailty was assessed using the Comprehensive Geriatric Assessment Frailty Index (CGA-FI). Sarcopenia was diagnosed based on the CT-based skeletal muscle index (SMI), grip strength, and physical function. The primary outcome was worsening disability or death within 3 months after surgery.A multivariate logistic regression model was used to estimate the association of sarcopenia and/or frailty with worsening disability or death within 3 months after surgery.

**Results:**

Patients with sarcopenia and frailty did not experience a reduction in disability burden within 3 months postoperatively [−2.0 (IQR: −9.0, 21.2)]. In contrast, patients with sarcopenia only and those with frailty only had significant reductions in the overall disability burden [−5.0 (IQR: −7.0, 0.0) and −5.0 (IQR: −8.0, −1.0)]. Compared with normal patients, those with both sarcopenia and frailty (OR = 3.09; 95% CI 1.61–5.96), those with sarcopenia only (OR = 2.65; 95% CI 1.38–5.12), and those with frailty only (OR 1.93; 95% CI 1.01–3.72) had a significantly increased risk of worsening disability or death within 3 months post-surgery.

**Conclusion:**

Frailty, sarcopenia, and their combination are associated with a significantly increased risk of worsening disability or death within 3 months after surgery. Notably, compared with patients with both sarcopenia and frailty, those with frailty alone or sarcopenia alone have a lower burden of postoperative disability.

## 1 Introduction

Delivering safe perioperative care and enhancing postoperative quality of life are core priorities for clinicians worldwide. Owing to the aging population worldwide, the needs of older patients are exerting an increasingly profound impact on the realisation of this goal [1]. Elderly individuals with cardiac disease face particularly daunting challenges, as they often exhibit a markedly reduced in physiological reserve, resulting in frailty and sarcopenia [2]. When coupled with the inherent high risks of cardiac surgery, these factors substantially increase the risk of postoperative complications and adverse clinical outcomes [3,4].

Skeletal muscle is the most abundant tissue in the human body. With advancing age, skeletal muscle function progressively declines [5]. Sarcopenia is characterized by the gradual loss of muscle mass and strength, which impairs postoperative functional recovery and increases mortality in older adults [6]. Frailty is recognized as a multidimensional syndrome that involves the culmination of age and disease-related deficits. Evidence-based studies have shown that frail elderly individuals are more vulnerable to the stress of anaesthesia and surgery, leading to inferior postoperative functional recovery [7,8]. In summary, sarcopenia is primarily centred on skeletal muscle dysfunction, whereas frailty is a broader construct that includes physical impairment, comorbidities, and psychosocial and cognitive domains. Although frailty and sarcopenia overlap, existing evidence suggests that they may occur independently and do not simply represent an inclusive relationship [[9], [10], [11]].

Previous studies on patients undergoing cardiac surgery have revealed that sarcopenia and frailty are associated with poor postoperative outcomes. However, few investigations have explored the combined effect of these two conditions on postoperative prognosis, and most existing studies in this field are retrospective or pilot designs with small sample sizes [4,12,13]. On the other hand, prior studies have focused predominantly on postoperative endpoints such as major complications and mortality. However, with improved living standards, older adults are no longer solely concerned with major surgical risks; they also have a growing and urgent demand for favourable functional recovery and improved quality of life after surgery. Accordingly, we performed a prospective cohort study to assess the value of sarcopenia and frailty in predicting short-term functional recovery in elderly patients undergoing cardiac surgery. We hypothesized that patients with either sarcopenia, frailty, or both are at greater risk of poor short-term functional recovery after surgery.

## 2 Methods

### 2.1 Study design and participants

This single-centre, cohort study was registered with the China Clinical Trials Registry (ChiCTR2400086075) and approved by the Ethics Committee of the Affiliated Hospital of Xuzhou Medical University (XYFY2024-KL170-01). The study was conducted in accordance with the STROBE guidelines (Strengthening the Reporting of Observational Studies in Epidemiology).

Patients aged ≥60 years who were scheduled to undergo elective open‑heart surgery under general anaesthesia were eligible for enrolment. All patients were required to undergo abdominal CT scanning within 90 days prior to surgery. The exclusion criteria were as follows: inability to understand the study‑related assessment scales or refusal to participate in the study; inability to complete the physical function assessment because of preoperative conditions, including osteoarthritis, lower extremity fractures, limb disabilities, or lower extremity/atrial thrombosis; inability to be contacted via telephone; and failure to complete the WHODAS assessment.

### 2.2 Clinical variables

Clinical baseline data, including sex, age, smoking status and alcohol consumption, comorbidities (including diabetes, hypertension, cerebral infarction, and myocardial infarction), surgical type, ASA classification, depression [14], cognitive impairment [15], pulmonary function (FEV1/FVC), and left ventricular ejection fraction (LVEF), as well as other relevant indicators, were collected for all the patients.

### 2.3 Frailty assessment

In this study, a widely used frailty assessment tool, the CGA‑FI, was used [16,17]. The CGA‑FI consists of 48 deficit variables covering chronic comorbidities, physical function, cognitive status, and psychological well‑being. The CGA‑FI score was calculated as the total sum of the deficit scores divided by 48 (range: 0–1; higher scores represent greater frailty severity). A score ≥0.25 was defined as frailty.

### 2.4 Sarcopenia assessment

Sarcopenia is defined as an age-related loss of skeletal muscle mass and strength, accompanied by and/or a decline in physical performance [18]. Skeletal muscle area was quantified at the level of the third lumbar vertebra (L3), which lies superior to the iliac crest. Abdominal CT data were extracted from routine preoperative full-length aortic computed tomography angiography (CTA) scans. These scans are performed to exclude aortic dissection and aneurysms and assess aortic calcification, providing critical information that guides surgeons in evaluating surgical feasibility and selecting the optimal surgical approach [19].These measurements were obtained by analysing abdominal computed tomography (CT) images using picture archiving and communication system (PACS) software, with a maximum interval of 3 months between CT image acquisition and the date of surgery. At the L3 level, skeletal muscle was segmented using a Hounsfield unit (HU) threshold ranging from −29 to +150. The L3 skeletal muscle area was manually delineated via freehand tracing (Supplementary Figure S1) and subsequently normalized for height to derive the skeletal muscle index (SMI, cm²/m²) [20].Low muscle mass was defined as an SMI <45 cm²/m² in men and <38 cm²/m² in women [12]. All CT image analyses and processing were conducted by a single investigator who was blinded to postoperative clinical outcomes.

Muscle strength was assessed using a hand dynamometer, with the maximum value recorded from two trials. Low muscle strength was defined as a grip strength of less than 18 kg for women and less than 28 kg for men [18].

Reduced physical function was defined as a walking speed of less than 1.0 m/s or a completion time of more than 12 seconds in the 5-time chair stand test. Walking speed is measured by recording the time to walk 5 metres at a normal pace, starting from movement initiation. The 5-time chair stand test was performed with the arms crossed over the chest, and the time required to complete five consecutive sit-to-stand repetitions was documented [18].

### 2.5 Sarcopenia frailty score

To better quantify the value of frailty and sarcopenia in predicting postoperative functional recovery in elderly patients undergoing cardiac surgery, we propose a new risk assessment indicator, the“Sarcopenia frailty score.” The score for this new risk assessment system ranges from 0 to 2 points, with 0 points indicating a normal individual, 1 point indicating the presence of frailty or sarcopenia alone, and 2 points indicating the presence of both frailty and sarcopenia.

### 2.6 Measurement of disability

The World Health Organization Disability Assessment Schedule 2.0 (WHODAS) is a validated instrument for evaluating disability. It assesses the severity of difficulty patients experienced over the past week in specific functional domains due to health problems, with responses graded on a 4-point scale: None = 0; Mild = 1; Moderate = 2; Severe = 3. The total score of the 12 items ranges from 0 to 48, which is then converted to a percentage by dividing by 48 and multiplying by 100, yielding a score from 0 (indicating no disability) to 100 (representing maximal disability or death). Both disability scores and the proportion of survivors with a disability-free status have been proposed as patient-centred perioperative outcome measures.

Distinct disability states are defined as follows [3,21,22]:

1) Minimum clinically important difference: an increase in the WHODAS score of 5% or greater;
2) Disability-free survival: a WHODAS score less than 25%;
3) Clinically significant disability: a WHODAS score of 35% or higher;
4) Disability worsening: a WHODAS score ≥25% with an absolute increase of ≥5%.

### 2.7 Outcomes

The primary outcome is worsening disability or death at 3 months.

Secondary outcomes included (1) patient-reported functional disability scores at baseline and at 1 month, and 3 months post-surgery; (2) disability-free survival rates at 1 month and 3 months post-surgery; (3) incidence of clinically significant disability at 1 and 3 months post-surgery; (4) patients’ place status at 1 and 3 months post-surgery; (5) incidence of PPCs [23]; (6) incidence of delirium during the postoperative hospital stay [24]; and (7) other major morbidities (wound infection, acute kidney injury, acute heart failure, second operation, and new-onset stroke) (Supplementary Table S1) [25].

### 2.8 Sample size

In our pilot cohort of 100 elderly patients who underwent elective open‑heart surgery, the incidence of worsening disability or death at 3 months post-surgery was approximately 20%. The sample size calculation was based on the expected postoperative event rate and the rule of thumb of at least 10 outcome events per predictor variable [26]. Assuming an event rate of 20% and 10 predictor variables, we aimed to recruit 500 patients. Accounting for an anticipated 15% dropout rate, the total target sample size was set at 588 patients.

### 2.9 Statistical analysis

Histograms were employed to examine variable distributions and assess normality. Normally distributed continuous variables are expressed as the mean ± standard deviation (mean ± SD), while non-normally distributed continuous data are reported as the median (interquartile range, IQR). Categorical data are presented as frequencies (%). Group comparisons for continuous variables were conducted using the independent-samples Student’s t-test or Mann–Whitney U test according to the data distribution. Categorical variables were analysed via the chi-square test or Fisher’s exact test, as applicable. When statistically significant differences were detected among multiple groups, post-hoc testing with the Bonferroni correction was performed.

No data regarding functional recovery within 90 days were deleted; the remaining missing data were imputed using multiple imputation methods.

Before constructing the multivariate logistic regression model, we first performed least absolute shrinkage and selection operator (LASSO) regression to select candidate covariates from variables that showed statistical significance in the univariate analysis, with the aim of retaining features with non-zero coefficients (Supplementary Fig. S2). The optimal penalty parameter included six covariates: sarcopenia frailty score, age, stroke/TIA, depression, diabetes, and NT-proBNP (Supplementary Table S2). In addition, sex was included as a baseline confounder based on the previous literature and its well-established clinical relevance. Therefore, to examine the associations between preoperative frailty and/or sarcopenia and worsening disability or death at 1 and 3 months, the sarcopenia frailty score was incorporated into the model as the primary exposure variable. Variables identified via LASSO regression were subsequently included as covariates in the multivariate logistic regression model to adjust for potential confounding factors. In addition, a multi-group propensity score-weighted analysis was conducted to account for observed potential confounding variables and to further validate the robustness of the primary outcome. Spearman’s rank correlation test was used to evaluate the correlations of the CGA-FI score with grip strength, gait speed, and skeletal muscle index. The correlation coefficients (*r*) were interpreted as weak (0.01–0.39), moderate (0.40–0.69), and strong (0.70–1.0).

Diagnostic performance was evaluated using receiver operating characteristic (ROC) curves. The area under the curve (AUC), quantified via the C-statistic, was computed to assess the predictive accuracy of the logistic models for the primary outcome. Model 1 served as the baseline risk model. The DeLong test was applied to compare the AUC values between the baseline risk model and the model incorporating the sarcopenia frailty score. Furthermore, the net reclassification index (NRI) and integrated discrimination improvement (IDI) were calculated to further evaluate the incremental predictive value of frailty and sarcopenia scores over established risk factors. A calibration curve was plotted to evaluate the calibration accuracy of the model.

As an exploratory analysis, we employed group-based trajectory modelling (GBTM) to identify distinct patient subgroups with similar disability trajectories over the 90-day postoperative period. We evaluated the models specifying two, three, and four potential trajectory classes. The best-fitting model was selected based on the lowest Akaike information criterion (AIC), and the clinical validity of the identified trajectory groups was confirmed.

All analyses were performed using R Statistical Software (Version 4.2.2; http://www.R-project.org; The R Foundation) and the Free Statistics analysis platform (Version 2.2; Beijing, China). A p-value < 0.05 (two-tailed) was considered to indicate statistical significance.

## 3 Results

### 3.1 Baseline characteristics

From June 30, 2024, to April 5, 2026, a total of 683 patients underwent surgery.Among these patients, 160 met the exclusion criteria. Ultimately, 523 patients were included in the final analysis (Fig. 1).

**Fig. 1:**
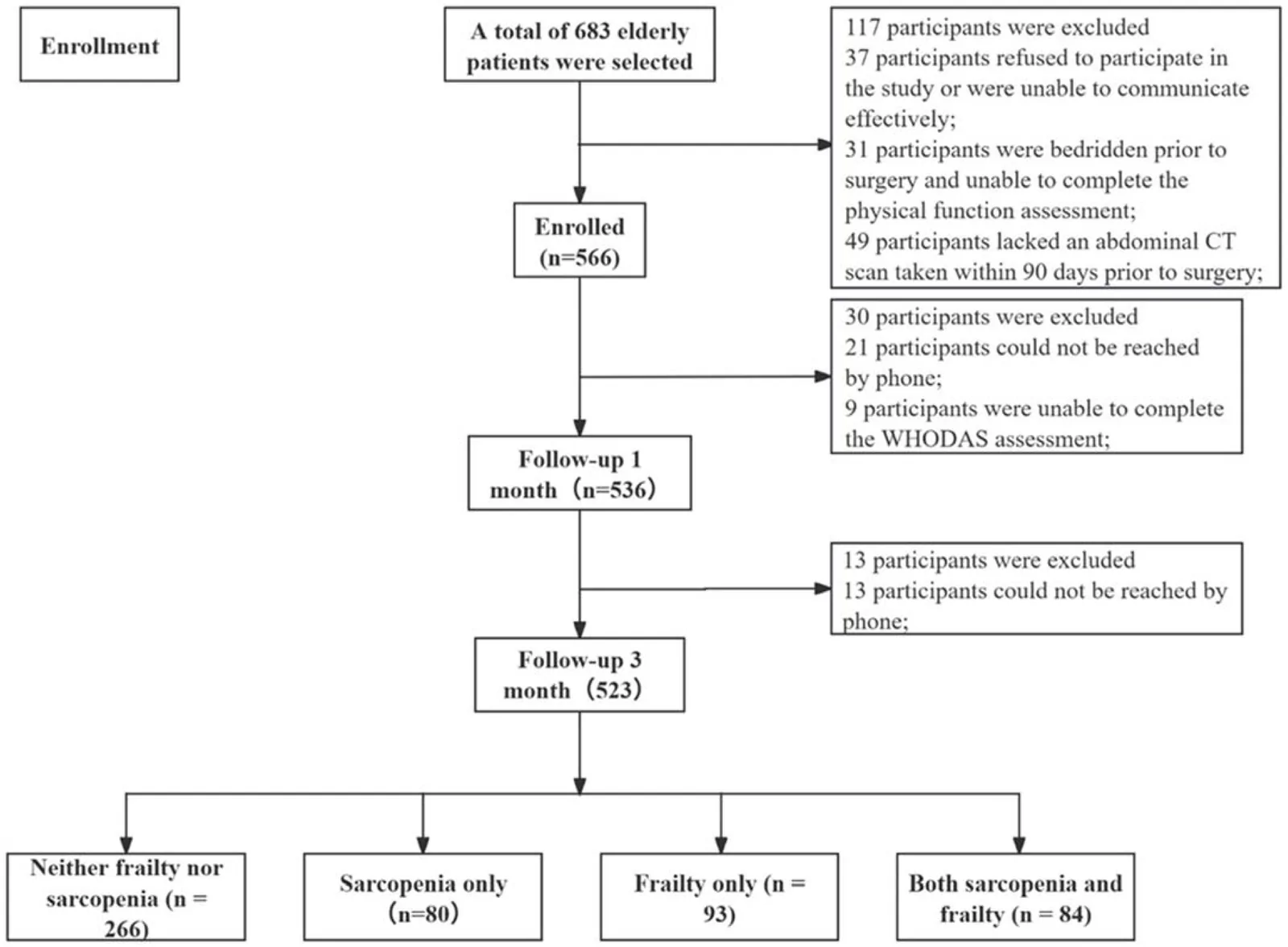
Study participant flow diagram.

Table 1 summarizes the baseline characteristics of the groups stratified by sarcopenia and frailty. Among the study population, the prevalence of sarcopenia was 31.4%, that of frailty was 33.8%, and the prevalence of concurrent sarcopenia and frailty was 16.1%. Compared with normal participants, patients with frailty and/or sarcopenia were generally older, had a lower body weight, and were more frequently female. In addition, patients in this group had a significantly higher prevalence of comorbidities, including diabetes, stroke/TIA, depression, and cognitive impairment. They also exhibited elevated levels of fasting blood glucose, NT-proBNP and blood urea nitrogen, as well as reduced levels of haemoglobin and albumin (all P < 0.001). Regarding physical function assessments, patients with sarcopenia and/or frailty had longer five‑chair rise times; higher EuroSCORE II, ASA, and NYHA classifications; and lower grip strength, walking speed, and skeletal muscle index. However, no significant differences were observed among the four groups in terms of alcohol consumption, hypertension, surgical type, serum creatinine, or operative duration (P > 0.008).

**Table 1:** Baseline characteristics.

Variables	Neither frailty nor sarcopenia (n = 266)	Sarcopenia only (n = 80)	Frailty only (n = 93)	Both sarcopenia and frailty (n = 84)	P
Age, Mean ± SD	65.7 ± 5.1	67.7 ± 5.5	67.9 ± 5.1	68.7 ± 5.8	<.001
Female, n(%)	78 (29.3)	37 (46.3)	36 (39.1)	47 (55.9)	<.001
BMI, Mean ± SD	24.7 ± 3.3	22.9 ± 2.6	25.9 ± 3.6	23.2 ± 3.5	<.001
Smoker, n(%)	106 (39.8)	16 (20.0)	32 (34.4)	24 (28.5)	0.007
Alcohol consumption, n(%)	70 (26.3)	13 (16.2)	27 (29.0)	17 (20.2)	0.152
Hypertension, n(%)	120 (45.1)	34 (42.5)	63 (67.7)	43 (51.2)	0.001
Diabetes, n(%)	55 (20.6)	10 (12.5)	37 (40.2)	27 (32.1)	<.001
Stroke/TIA, n(%)	101 (37.9)	34 (42.5)	54 (58.1)	47 (55.9)	0.001
MI, n(%)	19 (7.1)	7 (8.7)	14 (15.1)	19 (22.6)	<.001
HF, n(%)	17 (6.4)	9 (11.3)	13 (13.9)	17 (20.2)	0.003
AF, n(%)	31 (11.6)	21 (26.3)	11 (11.8)	13 (15.5)	0.011
PAH, n(%)	54 (20.3)	19 (23.7)	16 (17.2)	23 (27.4)	0.360
PCI, n(%)	18 (6.8)	7 (8.8)	10 (10.7)	3 (3.6)	0.291
CKD, n(%)	7 (2.6)	2 (2.5)	4 (4.3)	7 (8.3)	0.132
Bronchial disease, n(%)	14 (5.3)	4 (5.0)	13 (13.9)	13 (15.5)	0.003
Cough and sputum, n(%)	37 (13.9)	10 (12.5)	23 (24.7)	17 (20.2)	0.055
Depression, n(%)	7 (2.6)	3 (3.7)	10 (10.7)	17 (20.2)	<.001
Cognitive Impairment, n(%)	25 (9.4)	7 (8.7)	11 (11.8)	25 (29.7)	<.001
LVEF, Mean ± SD	58.9 ± 7.8	58.3 ± 8.0	57.8 ± 8.1	57.6 ± 8.2	0.455
FEV1/FVC, Mean ± SD	87.6 ± 12.5	86.6 ± 11.1	87.3 ± 14.2	85.3 ± 13.0	0.566
Albumin, Mean ± SD	42.4 ± 3.6	41.8 ± 3.3	40.7 ± 3.3	41.3 ± 3.6	<.001
NT-proBNP	255.5 (99.2,643.0)	342.5 (144.5,1172.0)	288.0 (102.0,764.50)	631.0 (252.0,1517.0)	<.001
Blood glucose, Mean ± SD	5.6 ± 1.3	5.5 ± 1.3	6.0 ± 1.6	6.3 ± 2.6	0.001
BUN, Mean ± SD	6.2 ± 2.8	6.1 ± 2.2	6.3 ± 2.5	7.4 ± 4.1	0.002
Haemoglobin, Mean ± SD	138.8 ± 15.2	132.2 ± 12.4	129.8 ± 15.8	124.5 ± 16.3	<.001
SCR, Mean ± SD	65.0 ± 16.5	61.6 ± 16.0	68.0 ± 21.1	68.9 ± 27.9	0.060
ALT, M (Q₁, Q₃)	22.0 (17.0,31.0)	21.0 (15.0,29.5)	21.0 (16.0,34.0)	22.0 (16.0,35.2)	0.756
AST, M (Q₁, Q₃)	22.0 (16.0,30.0)	21.0 (16.0,27.5)	23.0(15.0,33.0)	22.0 (15.5,32.5)	0.788
SMI, M (Q₁, Q₃)	49.2 (45.6,53.0)	37.9 (35.1,42.5)	49.4 (45.2,54.6)	37.5 (35.1,42.0)	<.001
Grip strength, Mean ± SD	30.4 ± 7.3	24.2 ± 7.3	26.6 ± 8.4	21.20 ± 6.4	<.001
5-metre walk, Mean ± SD	4.7 ± 0.9	5.4 ± 0.8	5.9 ± 2.1	6.6 ± 1.7	<.001
5TSTS, Mean ± SD	10.0 ± 2.1	11.2 ± 2.0	12.5 ± 3.5	14.6 ± 11.4	<.001
EuroSCORE II, M (Q₁, Q₃)	1.1 (0.8,1.4)	1.2 (0.9,1.6)	1.5 (1.1,2.0)	1.8 (1.4,2.5)	<.001
ASA, n(%)					<.001
2	10 (3.7)	5 (6.2)	2 (2.1)	0 (0.0)	
3	245 (92.1)	68 (85.0)	65 (69.9)	44 (52.4)	
4	11 (4.1)	7 (8.7)	26 (27.9)	40 (47.6)	
NYHA, n(%)					<.001
2	214 (80.4)	54 (67.5)	48 (51.6)	23 (27.4)	
3	51 (19.1)	26 (32.5)	41 (44.1)	60 (71.4)	
4	1 (0.4)	0 (0.0)	4 (4.3)	1 (1.2)	
Type of operation, n(%)					0.008
CABG	152 (57.1)	34 (42.5)	64 (68.8)	43 (51.2)	
Valve	94 (35.3)	39 (48.7)	19 (20.4)	35 (41.6)	
CABG + Valve	17 (6.4)	4 (5.0)	8 (8.6)	5 (5.9)	
Other	3 (1.1)	3 (3.7)	2 (2.1)	1 (1.2)	
Off-pump surgery, n(%)	26 (9.8)	4 (5.0)	13 (13.4)	8 (9.5)	0.267
Bypass time(h), M (Q₁, Q₃)	1.7(1.3,2.1)	1.7 (1.3,2.1)	1.6 (1.3,2.3)	1.6 (1.3,2.1)	0.946
Surgery duration (h), M (Q₁, Q₃)	4.8 (4.0,5.6)	4.6 (3.6,5.43)	5.22 (4.3,6.0)	4.7 (4.1,5.5)	0.018

Data: Means ± standard deviations, absolute rates, and percentages. Statistical significance was defined as a p value< 0.05. BMI, body mass index; TIA, transient ischaemic attacks;MI, myocardial infarction; HF, heart failure; AF, atrial fibrillation; PAH, pulmonary arterial hypertension; PCI, percutaneous coronary intervention; CKD, chronic kidney diseases; LVEF, left ventricular ejection fraction; FVC, forced vital capacity; FEV1, forced expiratory volume In 1 s; NT-proBNP, N-terminal pro-B-type natriuretic peptide; BUN, blood urea nitrogen; SCR, serum creatinine; ALT, alanine aminotransferase; AST,aspartate sminotransferase; SMI, skeletal muscle index; 5TSTS, 5 Times Sit-to-Stand Test; EuroSCORE II, European System for Cardiac Operative Risk Evaluation II; ASA, American Society of Anesthesiologists; NYHA, New York Heart Association; CABG, coronary artery bypass graft.

### 3.2 The association of sarcopenia or (and) frailty with postoperative outcomes

Table 2 shows the proportions of patients who achieved functional recovery within 30 or 90 days after surgery across the groups. Compared with normal patients, those with sarcopenia or (and) frailty had significantly higher median [IQR] perioperative disability scores, higher rates of worsening disability or death, and higher rates of clinically significant functional decline, while their rates of disability-free survival were lower (P < 0.001).

**Table 2:** Postoperative functional recovery and major complications in patients with frailty and sarcopenia.

Variables	Neither frailty nor sarcopenia (n = 266)	Sarcopenia only (n = 80)	Frailty only (n = 93)	Both sarcopenia and frailty (n = 84)	P
Disability scores [IQR],(WHODAS)					
Preoperative	4.0 (2.0,7.0)	11.0 (9.0,12.0)	13.0 (7.0,18.0)	15.0(10.7,19.3)	<.001
1Month	7.0 (4.0,12.0)	10.0 (6.0,20.0)	12.0 (4.0,22.0)	17.5(12.0,40.0)	<.001
3Months	2.0 (0.0,5.7)	5.00 (4.0,12.3)	6.0 (2.0,14.0)	11.0 (4.0,42.0)	<.001
Change in disability score relative to baseline [IQR]					
1 Months	3.0 (1.0, 6.0)	1.0 (−5.0,7.2)	−1.0 (−4.0,6.0)	4.0 (−2.0,19.2)	<.001
3 Months	−1.0 (−3.0, 0.0)	−5.0 (−7.0,0.0)	−5.0 (−8.0,-1.0)	−2.0 (−9.0,21.2)	<.001
Worsening disability or death, n (%)					
1 Month	77 (28.9)	34 (42.5)	37 (39.7)	43 (51.2)	0.001
3 Months	29 (10.9)	21 (26.2)	22 (23.6)	29 (34.5)	<.001
Clinically significant disability, n (%)					
1 Month	28 (10.5)	26 (32.5)	38 (40.8)	42 (50.0)	<.001
3 Months	17 (6.4)	13 (16.2)	19 (20.4)	32 (38.1)	<.001
Disability-free survival(DFS), n (%)					
1 Month	193 (72.5)	42 (52.5)	43 (46.2)	24 (28.5)	<.001
3 Months	239 (89.8)	59 (73.7)	64 (68.8)	49 (58.3)	<.001
Place in 1 month, n(%)					<.001
Home	232 (87.2)	67 (83.7)	67 (72.0)	53 (63.1)	
Follow-up care or rehabilitation	9 (3.4)	4 (5.0)	6 (6.4)	3 (3.6)	
Medical wards	15 (5.6)	4 (5.0)	10 (10.7)	9 (10.7)	
ICU	4 (1.5)	2 (2.5)	6 (6.4)	7 (8.3)	
Dead	6 (2.2)	3 (3.7)	4 (4.3)	12 (14.3)	
Place in 3 months, n(%)					<.001
Home	242 (90.9)	70 (87.5)	74 (79.5)	59 (70.2)	
Follow-up care or rehabilitation	12 (4.51)	5 (6.2)	5 (5.4)	5 (5.9)	
Medical wards	4 (1.5)	1 (1.2)	4 (4.3)	2 (2.4)	
ICU	0 (0.0)	0 (0.0)	1 (1.1)	1 (1.2)	
Dead	8 (3.0)	4 (5.0)	9 (9.7)	17 (20.2)	
ICU stay, M (Q₁, Q₃)	17.5 (17.0,19.4)	19.0 (17.0,21.0)	18.0 (17.0,34.0)	19.3 (18.0,51.0)	<.001
Length of stay, M (Q₁, Q₃)	9.0 (8.0,11.0)	10.0 (8.0,12.0)	10.0 (8.0,13.0)	11.0 (9.0,14.0)	<.001
In hospital mortality, n(%)	5 (1.8)	2 (2.5)	5 (5.4)	14 (16.7)	<.001
Second operation, n(%)	14 (5.2)	5 (6.2)	4 (4.3)	7 (8.3)	0.657
Readmission in 90 days, n(%)	19 (7.1)	7 (8.7)	17 (18.3)	12 (14.3)	0.013
AHF, n(%)	11 (4.1)	7 (8.7)	9 (9.7)	20 (23.8)	<.001
New stroke, n(%)	4 (1.5)	0 (0.0)	2 (2.1)	0 (0.0)	0.481
Delirium, n(%)	22 (8.3)	8 (10.0)	16 (17.2)	26 (30.9)	<.001
PPCs, n(%)	58 (21.8)	24 (30.0)	46 (49.4)	45 (53.6)	<.001
Pulmonary infection, n(%)	30 (11.3)	10 (12.5)	22 (23.6)	24 (28.6)	<.001
AKI, n(%)	28 (10.5)	12 (15.0)	20 (21.5)	24 (28.6)	<.001
Wound infection, n(%)	9 (3.4)	3 (3.7)	6 (6.4)	7 (8.3)	0.221

WHODAS, WHO Disability Assessment Schedule 2.0; DFS, disability-free survival; IQR, interquartile range; ICU, intensive care unit; AHF, acute heart failure; PPCs, postoperative pulmonary complications; AKI, acute kidney injury.

There were significant differences in the incidence of major adverse events among the four groups (P < 0.001), and there were also statistically significant differences in the rates of home discharge within 90 days postoperatively, PPCs, AKI, delirium, AHF, and the length of hospital stay (P < 0.001). Furthermore, there were no significant differences among the groups in terms of the rate of second operation, wound infection, 90-day readmission, or new-onset stroke (P > 0.008).

### 3.3 Postoperative disability score trajectories in sarcopenic and frail patients

The median disability score in patients with frailty alone or sarcopenia alone did not decrease significantly from baseline to postoperative month 1 (P > 0.05), but decreased significantly by postoperative month 3 (P < 0.001). In contrast, the disability score in healthy patients increased significantly by 1 month (P < 0.001) but then decreased significantly by 3 months (P < 0.001). Patients with concurrent sarcopenia and frailty exhibited a postoperative trajectory similar to that of normal patients; however, they continued to carry a substantial disability burden in the early postoperative period and failed to achieve the primary goal of reducing this burden (Table 2 and Fig. 2). The minimum clinically significant change in disability score was defined as a 5% relative change (equivalent to 3 points). Compared with baseline data, the overall short-term disability burden in patients with combined sarcopenia and frailty did not decrease within 3 months postoperatively [median change: −2.0 (IQR: −9.0, 21.2)]. In contrast, the overall disability burden decreased significantly in patients with sarcopenia only [median change: −5.0 (IQR: −7.0, 0.0)] and in those with frailty only [median change: −5.0 (IQR: −8.0, −1.0)]. The change in scores in the control group was not statistically significant [median change: 2.0 (interquartile range: 0.0, 5.7)], and these patients had lower overall disability scores postoperatively (Table 2).

**Fig. 2:**
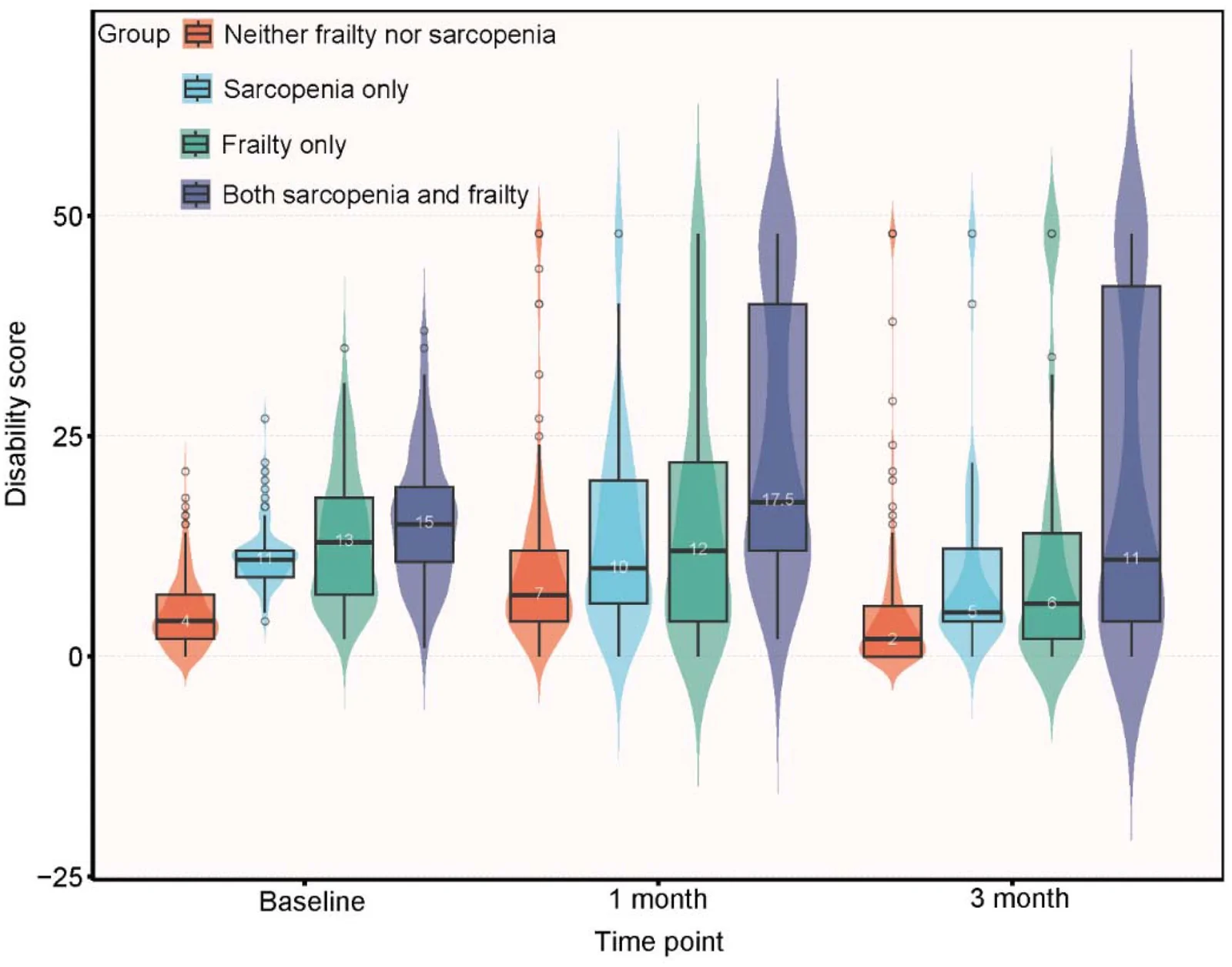
The World Health Organization Disability Assessment Scale 2.0 (WHODAS 2.0) was used to assess functional disability, with scores ranging from 0 to 48, where higher scores indicate greater disability severity. Statistically significant between-group differences in disability levels were observed in the open surgery cohort at both the 30-day and 90-day follow-up assessments.

### 3.4 Risk factors associated with worsening disability or death within 90 days post-surgery

As shown by the least absolute shrinkage and selection operator (LASSO) regression analysis (Supplementary Fig. S2), six variables with non-zero coefficients were retained in the final model, namely NT-proBNP, age, depression, sarcopenia frailty score, stroke/TIA, and diabetes (Supplementary Table S2). Table 3 shows that in the multivariate analysis, the sarcopenia frailty score was treated as the primary exposure variable, and its association with major postoperative worsening disability or death remained significant after adjusting for NT-proBNP, age, depression, stroke/TIA, and diabetes. In the fully adjusted model (Model 3), compared with normal patients, sarcopenia concurrent with frailty (OR, 3.09; 95% CI, 1.61–5.96), sarcopenia alone (OR, 2.65; 95% CI, 1.38–5.12), and frailty alone (OR, 1.93; 95% CI, 1.01–3.72) were independently associated with worsening disability or death within 3 months postoperatively (P < 0.05).

**Table 3:** Association of frailty and sarcopenia with worsening disability or death in a multiple regression model.

Variables	Model 1		Model 2		Model 3	
	OR (95% CI)	P	aOR (95% CI)	P	aOR (95% CI)	P
1 month						
Neither frailty nor sarcopenia	1.00 (Reference)		1.00 (Reference)		1.00 (Reference)	
Sarcopenia only	1.81 (1.08∼3.04)	0.024	1.62 (0.95∼2.75)	0.074	1.61 (0.94∼2.76)	0.081
Frailty only	1.62 (0.99∼2.65)	0.054	1.47 (0.89∼2.45)	0.135	1.31 (0.77∼2.22)	0.320
Both sarcopenia and frailty	2.57 (1.56∼4.26)	<.001	2.15 (1.27∼3.64)	0.004	2.05 (1.17∼3.57)	0.012
3 month						
Neither frailty nor sarcopenia	1.00 (Reference)		1.00 (Reference)		1.00 (Reference)	
Sarcopenia only	2.91 (1.55∼5.46)	<.001	2.78 (1.46∼5.29)	0.002	2.65 (1.38∼5.12)	0.004
Frailty only	2.53 (1.37∼4.68)	0.003	2.33 (1.24∼4.41)	0.009	1.93 (1.01∼3.72)	0.049
Both sarcopenia and frailty	4.31 (2.38∼7.79)	<.001	3.95 (2.13∼7.35)	<.001	3.09 (1.61∼5.96)	<.001

Model 1: Unadjusted model. Model 2: Adjusted for sex and age. Model 3: Model 2 + NT-proBNP, depression, stroke/TIA, and diabetes.

In the multiple-group propensity score-weighted analysis, both sarcopenia and frailty remained independently associated with the composite endpoint of worsening disability or death within 90 days after surgery (Supplementary Table S3).

### 3.5 Predictive value of sarcopenia-frailty scores for worsening disability or death

In the analysis of elderly patients undergoing elective open-heart surgery, ROC curves were constructed to evaluate the predictive ability of the baseline risk model(which included NT-proBNP, age, stroke/TIA, and diabetes) and the baseline risk model combined with the sarcopenia frailty score for predicting short-term postoperative worsening disability or death (Fig. 3A). The C-statistic, NRI, and IDI are listed in Table 4. The results revealed that in elderly patients who underwent elective open-heart surgery, the sarcopenia-frailty score had a significant incremental effect on the predictive value of the baseline risk model (change in C-statistic: 0.05, P = 0.034; NRI: 0.467, P < 0.0001; IDI: 0.030 [0.0003, 0.0286], P = 0.0002). Furthermore, after the sarcopenia frailty score was incorporated, the predicted risk was well calibrated against the observed outcomes (Fig. 3B).

**Fig. 3:**
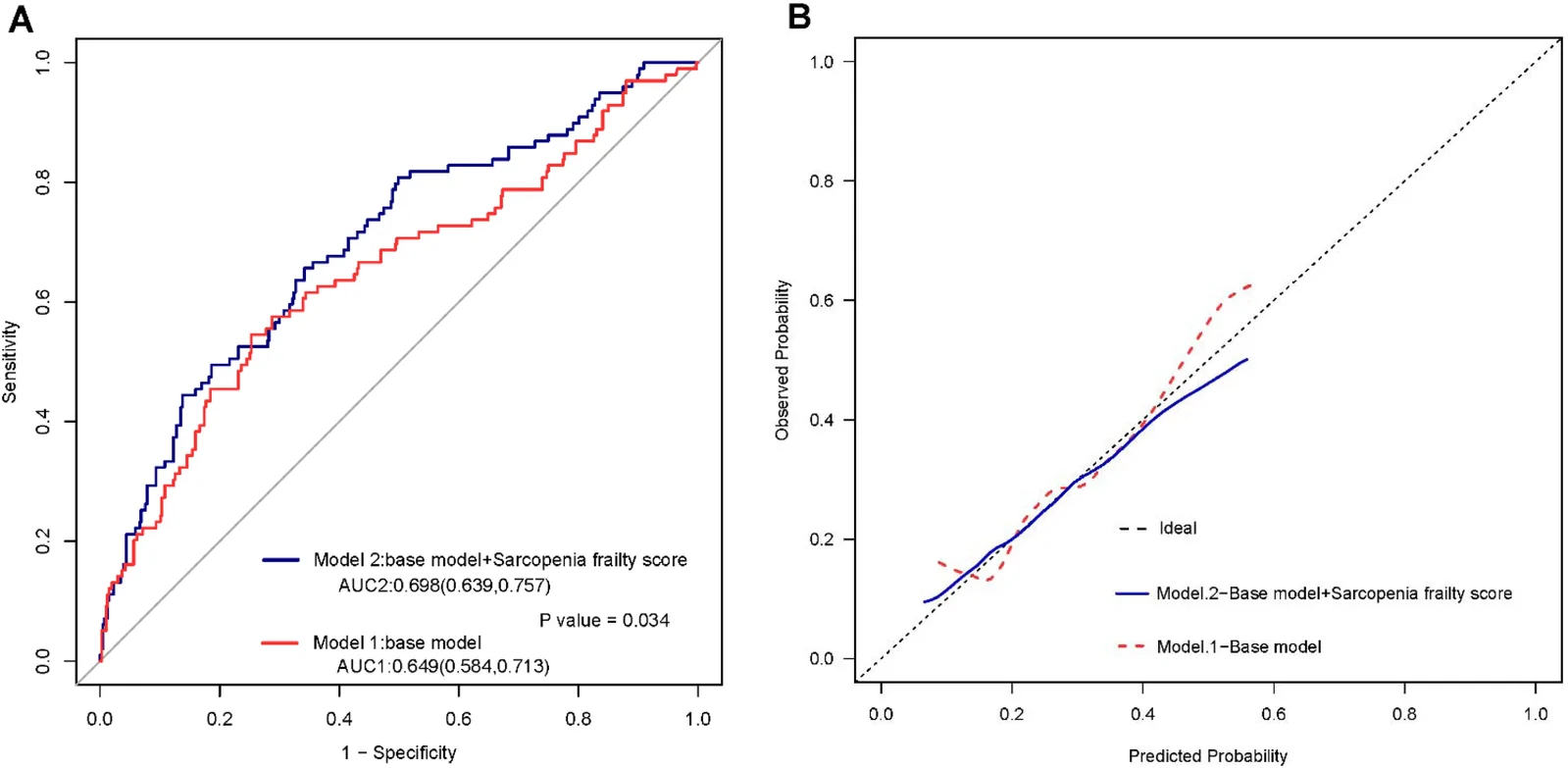
Receiver operating characteristic (ROC) curves for the predictive ability of the sarcopenia frailty score for postoperative worsening disability or death (A). Baseline risk model (Model 1) versus baseline risk model plus sarcopenia-frailty score (Model 2). The baseline risk model includes age, NT-proBNP, depression, stroke/TIA, and diabetes. A calibration curve was plotted to examine the relationship between observed risk (y-axis) and predicted risk (x-axis). In the figure, the dashed line represents ideal calibration; the closer the curve is to the dashed line, the better the calibration. Model 2 demonstrates that the baseline model, when combined with the sarcopenia-frailty score, yields better calibration results (B).

**Table 4:** Incremental predictive value and discriminative performance of various models assessed by C-statistics, NRI, and IDI.

Models	C statistics (95 % CI)	P	NRI (95% CI)	P	IDI (95 % CI)	P
Base model	0.648 (0.583∼0.713)	Ref.	Ref.		Ref.	
+sarcopenia frailty score	0.698 (0.639∼0.758)	0.034	0.467 (0.2561∼0.678)	<0.0001	0.030 (0.014∼0.046)	0.0002

The baseline risk model included age,NT-proBNP, depression, stroke/TIA, and diabetes. CI, confidence interval; IDI, integrated discrimination improvement; NRI, net reclassification improvement.

We next evaluated the incremental prognostic value of the sarcopenia frailty score compared with that of frailty alone and sarcopenia alone. ROC curve analysis demonstrated that the sarcopenia frailty score had the greatest discriminative power for the composite outcome of postoperative worsening disability or death, with an AUC of 0.694, compared with 0.654 for frailty alone and 0.619 for sarcopenia alone (Supplementary Fig. S3).

### 3.6 Association of the frailty index with perioperative grip strength, skeletal muscle index, and grip strength

Preoperative CGA-FI scores were weakly correlated with grip strength (*R* = −0.324; P < 0.001), and skeletal muscle index (*R* = −0.198; P < 0.001), and moderately correlated with walking speed (*R* = −0.462; P < 0.001) (see Supplementary Fig. S4).

### 3.7 Exploratory group-based trajectory models

The four-trajectory model best fit our data and was deemed clinically meaningful (Fig. 4). Model fitting statistics are presented in Supplementary Tables S4 and S5, demonstrating excellent model fit and clinical utility. In this elderly cardiac surgery population, two trajectory groups (representing approximately 77% of participants) showed a steady decline in disability levels from baseline to 3 months postoperatively. In contrast, the remaining approximately one-quarter of patients experienced an initial increase in disabilitywithin 1 month post-surgery, followed by a slow recovery, but continued to carry a substantial disability burden at the 3-month follow-up.

**Fig. 4:**
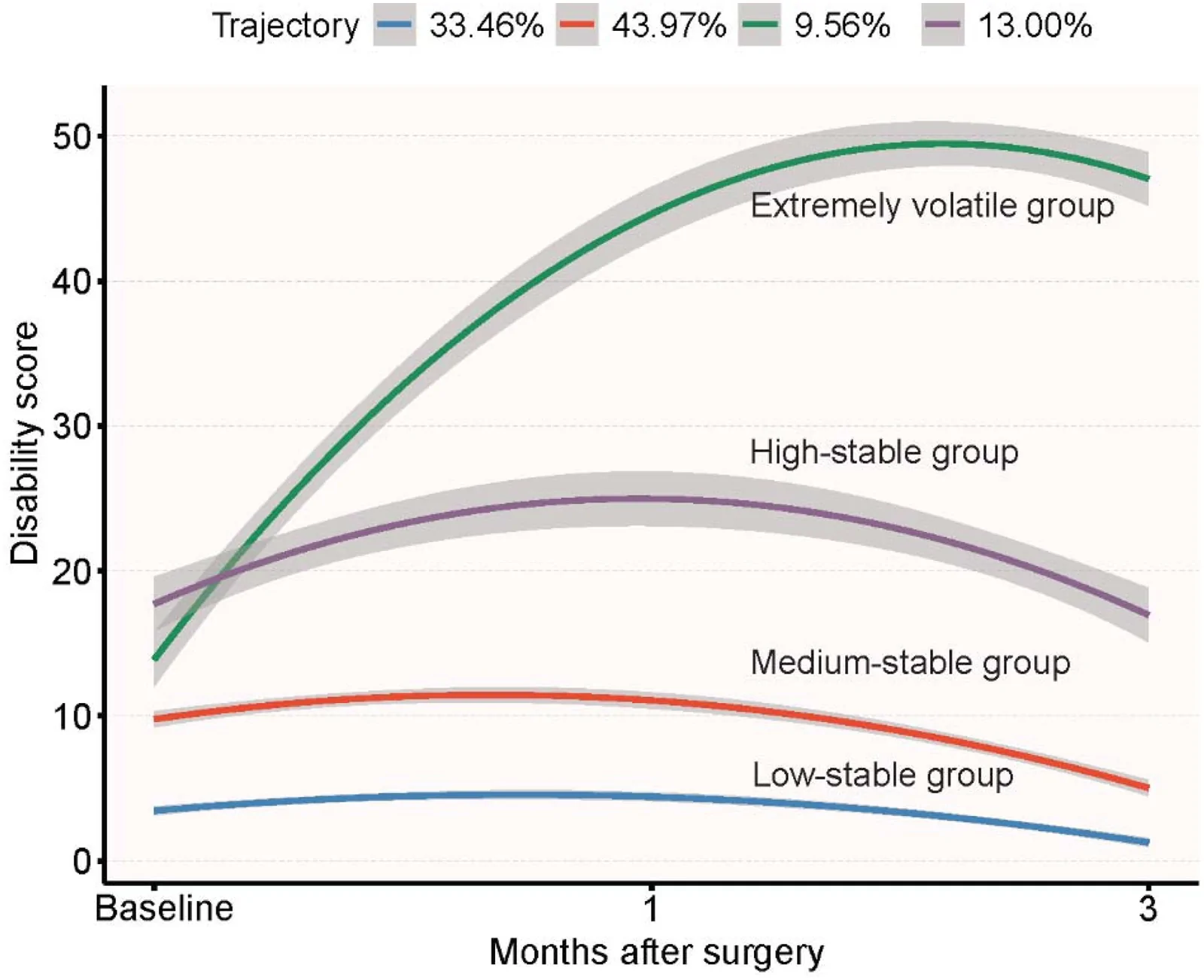
The WHODAS score trajectories and the proportion of participants in each group were as follows: low-stable trajectory (34.46%), medium-stable trajectory (43.97%), high-stable trajectory (13.00%), and extremely high-volatile group (9.56%). Each trajectory group is based on the best-fitting group trajectory model determined by the lowest akaike information criterion (AIC) value.

## 4 Discussion

In this prospective cohort study, we reported that frailty, sarcopenia, and the combination of sarcopenia and frailty were associated with a significantly increased risk of worsening disability or death within 3 months after surgery. Notably, compared with patients who had both sarcopenia and frailty, those with frailty alone or sarcopenia alone had a lower burden of postoperative disability. These novel patient-reported outcomes offer valuable insights to help perioperative physicians balance risks and benefits in surgical decision-making.

Our study demonstrated that sarcopenia is an independent risk factor for poor functional recovery after cardiac surgery in elderly patients, which is consistent with the findings reported in previous literature. A retrospective Korean study of 2,180 patients who underwent coronary artery bypass grafting (CABG) by Lee et al. revealed that decreased muscle mass defined by the computed tomography-derived skeletal muscle index (SMI), was associated with an increased long-term risk of mortality following CABG [12]. In a separate prospective, multicentre cohort study performed across Europe and the United States, Afilalo et al. further reported that sarcopenia identified on routine clinical CT scans was predictive of one-year mortality in patients who underwent transcatheter aortic valve replacement (TAVR) [27]. Although the evidence base from these studies is relatively robust, most investigations evaluating sarcopenia are retrospective in design, and reliance on the skeletal muscle index (SMI) alone fails to provide a comprehensive diagnosis of sarcopenia as defined by current international consensus criteria [18]. A clear conceptual distinction exists between dynapenia and skeletal muscle mass. Dynapenia, first proposed by Clark and Manini, specifically refers to agerelated declines in muscle strength; this phenotype can manifest independently of alterations in muscle mass [28]. According to the EWGSOP2 consensus [29], which recognizes muscle strength as having superior prognostic value over muscle mass and places it at the core of sarcopenia diagnosis, the incorporation of muscle strength into sarcopenia assessment markedly improves its performance in identifying perioperative risk. Furthermore, the outcome measures in these studies have predominantly focused on the incidence of major postoperative complications or all-cause mortality and therefore do not capture the full spectrum of patients’ postoperative experiences, including pain, depressive symptoms, health-related quality of life, and increased decisional regret.

Our study also revealed that frailty is closely associated with postoperative functional status, which is consistent with the findings reported in previous literature [7,30]. A prospective multicentre study of noncardiac surgery patients revealed that compared with nonfrail patients, frail patients had significantly more severe disability but showed substantial improvements in disability scores over time [31]. Another study with a short-term follow-up of 148 cardiac surgery patients revealed that although the burden of disability continued to decrease postoperatively in frail patients, nonfrail patients experienced worsening disability within 1 month after surgery but gradually recovered by 3 months [4]. A large-scale investigation further revealed that functional recovery following major elective noncardiac surgery generally requires 3 to 6 months. Within 6 months postoperatively, one in six patients experienced worsening disability or death, with preoperative frailty identified as a key risk factor [32].

Sarcopenia and frailty are both prevalent syndromes of physiological decline in older adult surgical patients. While the two conditions are strongly correlated, each condition presents distinct clinical and pathophysiological features [33]. The core distinction lies in their phenotypic focus: sarcopenia is defined by structural and functional impairments of the musculoskeletal system, with its diagnosis anchored to core metrics, including muscle mass, muscle strength, and physical performance [34]. In contrast, frailty is a multisystem, multidimensional syndrome of physiological vulnerability, which, beyond declines in muscle function, encompasses impairments across multiple domains, including psychological status, nutritional reserve, cognitive function, mood regulation, and comorbidity burden [[35], [36], [37]]. Critically, the relationship between these two syndromes is not a simple hierarchical one in which the latter encompasses the former. Consistent with prior evidence, our analyses revealed that the frailty index was weakly correlated with grip strength and the skeletal muscle index and moderately correlated with gait speed. These findings further support the notion that frailty and sarcopenia are interrelated but represent two distinct physiological states. Previously, a study by Guo et al. in patients undergoing noncardiac surgery demonstrated that sarcopenia and frailty are two distinct clinical phenotypes [11]. There was no significant interaction between these two conditions with respect to the risk of major postoperative complications; instead, they exerted independent effects with an additive effect on adverse outcomes. The study further confirmed that combined assessment of sarcopenia and frailty may offer superior clinical predictive value, a finding that aligns closely with the results of our present study [10,11].

In clinical practice, the concurrent identification and combined assessment of sarcopenia and frailty perioperatively provide critical guidance for clinical decision-making. Because single-domain assessments capture only partial features of either musculoskeletal function or global physiological status, perioperative risk may be underestimated; in contrast, combined assessments enable more comprehensive quantification of a patient’s overall physiological reserve and stress tolerance, thus more precise preoperative risk stratification and robust prediction of postoperative adverse outcomes can be achieved [38,39]. Notably, while combined assessments of sarcopenia and frailty incur additional time costs, this strategy delivers significant clinical benefits for high-risk older adult patients undergoing cardiac surgery and is therefore a necessary component of routine clinical assessment in this population.

Compared with previous similar studies in cardiac surgery patients, our work presents three key innovations and novel findings [4,12]. First, we adopted a patient-centred approach, focusing on patient-reported functional recovery outcomes, thereby addressing a key limitation of prior studies that predominantly centred on hard clinical endpoints such as mortality and perioperative adverse events. Second, in contrast to most prior investigations examining these two syndromes in isolation, our study systematically examined the combined effects of sarcopenia and frailty. Most importantly, by leveraging prospective follow-up data, we identified markedly divergent postoperative functional recovery trajectories between two patient subgroups: patients with comorbid sarcopenia and frailty exhibited a significant increase in disability scores at 1 month postoperatively compared to preoperative baseline scores, and failed to return to baseline levels by 3 months postoperatively, indicating a state of persistent functional disability. In contrast, patients with sarcopenia alone or frailty alone showed an overall downwards trend in disability scores at both 1 and 3 months post-surgery, indicating a gradual recovery of functional status. These completely divergent functional recovery trajectories directly validate the significant clinical value of a combined assessment for sarcopenia and frailty. Based on these findings, our study offers clear clinical implications: for patients with concurrent sarcopenia and frailty, surgery alone fails to achieve meaningful improvements in short-term postoperative quality of life. Therefore, for high-risk patients with this dual comorbidity, we outline three core interventional pathways that may mitigate perioperative frailty and improve functional outcomes: exercisebased prehabilitation to increase muscle strength and cardiopulmonary reserve; nutritional optimization to counteract muscle wasting; and integrated multidisciplinary geriatric rehabilitation pathways encompassing surgical, anaesthetic and nursing care [39].

Our study has several limitations. First, because this is an observational study, there may be unmeasured confounding factors; future research should involve multicentre studies with large sample sizes. Second, compared with traditional assessments, simultaneous assessment of frailty and sarcopenia may be time-consuming and less convenient for busyperioperative physicians; however, we believe that it is worthwhile for high-risk surgeries. Third, our inclusion of patients with preoperative abdominal CT scans may have introduced selection bias. In addition, the skeletal muscle index cut-off value used in this study has not been formally validated in large-scale, population-based studies of the local population. We therefore opted to use the cut-off value derived from a South Korean cohort, because of the shared Asian ethnicity and comparable body composition between the two populations. Fourth, the WHODAS 2.0 captures patient‑reported functional impairment. Nevertheless, the absence of objective performance‑based physical measures, including the SPPB, TUG, and 6MWT, limits a complete characterization of objective physical recovery. Thus, future studies should integrate patient‑reported questionnaires with objective physical performance tests to achieve a more comprehensive evaluation of postoperative functional recovery among older adults undergoing cardiac surgery. Finally, we tracked functional scores during the first 30 and 90 days after surgery; hence,the resulting postoperative functional recovery trajectories were incomplete and short-term. Long-term follow-up evaluations should be incorporated in future studies to further investigate the effects of sarcopenia and frailty on long-term functional recovery in elderly patients undergoing cardiac surgery.

## 5 Conclusion

Through comprehensive preoperative assessments, we found that frailty, sarcopenia, and their combination were associated with a significantly increased risk of worsening disability or death within 3 months after surgery. Notably, patients with frailty or sarcopenia alone had a lower burden of postoperative disability than patients with both conditions did. These findings suggest that comprehensive assessment of sarcopenia and frailty is crucial for perioperative risk stratification and prognostic evaluation.

## CRediT authorship contribution statement

**Guanglei Fan:** Data collection, Writing – original draft, Visualization, Supervision, Project, administration. **Yang Zhang:** Methodology, Investigation, Formal analysis, Conceptualization. **Guoqiang Chen:** Data collection, Supervision, Methodology, Investigation.**Wanzhou Wang:**Data curation, Conceptualization. **Wenjing Zhao:** Investigation, Data curation, Conceptualization. **Xiaowen Meng:** Writing – review & editing, Supervision; **Fuhai Ji:** Resources, Project administration, Methodology, Conceptualization.

## Ethical statement

This study was registered with the China Clinical Trials Registry (ChiCTR2400086075) and approved by the Ethics Committee of the Affiliated Hospital of Xuzhou Medical University (XYFY2024-KL170-01).

The study was conducted in accordance with the Declaration of Helsinki, and all participants signed a written informed consent form.

## Ethics declaration

The author(s) declare(s) that the study does not involve humans nor animal subjects.

## Declaration of Generative AI and AI-assisted technologies in the writing process

ChatGPT was used for grammar, spelling, and sentence structure assistance during manuscript preparation. The model did not generate any analytical or interpretive content. All outputs were reviewed and edited by the authors, who bear full responsibility for the final work.

## Funding

This work was supported by the 10.13039/501100001809National Natural Science Foundation of China (Grant Nos. 82471281 and 82302465), the Boxi Excellent Talent Cultivation Program (0302020201036), the Key Research and Development Program of Jiangsu Province (Grant No. BE2023709), the Suzhou Health Talent Program (Grant No. GSWS2022007), the Suzhou Key Laboratory of Anesthesiology (SZS2023013), the Suzhou Clinical Medical Center for Anesthesiology (Grant No. Szlcyxzxj202102), and the National Clinical Key Specialty of Anesthesiology.

## Data availability

The anonymized raw data supporting the findings of this study are available from the corresponding author, Fuhai Ji, upon reasonable request. The data are not publicly accessible because of privacy and ethical restrictions.

## Declaration of competing interest

The authors declare that they have no known competing financial interests or personal relationships that could have appeared to influence the work reported in this paper.
