# Supplementary material for: Impact of preoperative frailty and sarcopenia on short-term functional recovery after cardiac surgery in elderly patients: a prospective cohort study
Source: J Nutr Health Aging. 2026 Oct 9;30(12):101003. doi: 10.1016/j.jnha.2026.101003 (PMC13677951; doi:10.1016/j.jnha.2026.101003)
Supplement: Supplementary file 1 [file mmc1.docx]

Impact of preoperative frailty and sarcopenia on short-term functional recovery after cardiac surgery in elderly patients: a prospective cohort study

supplementary materials

Supplementary Figure 1: Representative examples of L3-level cross-sectional computed tomography images used to assess skeletal muscle mass in elderly cardiac surgery patients with (A) non-sarcopenia and (B) sarcopenia.

Supplementary Table 1. Major morbidity definition

Stroke: confirmed by CT brain results;
AKI: new requirement for dialysis or increase in serum creatinine >153 umol/l [>2mg/dl] and >2-fold the pre-operative level)

Wound infection: requirement for operative intervention and antibiotic therapy, with positive culture;

Pulmonary infection: new pulmonary infiltrate on chest x-ray plus at least two of the following: temperature > 38°5C or < 35.5°C, leukocytosis or leukopenia (white blood cells > 12,000 cells/mm3 or < 4000 cells/mm3), purulent secretions and antibiotic treatment;

Second operation: After the operation, due to bleeding during the thoracotomy or wound infection, a surgical procedure was performed under general anesthesia.

Delirium: disturbed state of consciousness and cognitive dysfunction with or without agitation.

AHF: The Framingham Heart Failure (FC-HF) diagnostic criteria comprise two categories: major and minor. Major criteria include paroxysmal nocturnal dyspnea, orthopnea, jugular venous distension, third heart sound, cardiothoracic ratio >0.5 on chest radiography, pulmonary edema, and pulmonary crackles. Minor criteria consist of peripheral edema, nocturnal cough, exertional dyspnea, hepatomegaly, pleural effusion, and tachycardia (heart rate ≥100 beats per minute). A diagnosis of FC-HF requires the presence of two major criteria or one major plus two minor criteria.

AKI, acute kidney injury. AHF, acute heart failure.

Supplementary Figure 2. LASSO regression analysis plots. (A) Partial likelihood deviance plot. The bold dashed line indicates the optimal λ value selected for the final model. (B) LASSO coefficient profile plot. Each curve represents the coefficient trajectory of an individual feature as a function of log(λ). At the optimal λ value of 0.017, 6 variables were retained in the model.

Supplementary Table 2 Coefficients and Lambda.1se value of the LASSO regression.

| Variable.name | Coefficient | lambda.type | lambda.value |
| --- | --- | --- | --- |
| NT-ProBNP | 0 | lambda.min | 0.017 |
| Age | 0.019 | lambda.min |  |
| Depression | 0.274 | lambda.min |  |
| Sarcopenia frailty score | 0.325 | lambda.min |  |
| Stroke/TIA | 0.045 | lambda.min |  |
| Diabetes | 0.121 | lambda.min |  |

Supplementary Table 3 Associations between sarcopenia, frailty, and worsening disability or death at 90 days in unadjusted, multivariate, and propensity score-adjusted analyses.

| Models | Variable | OR (95%CI) | P |
| --- | --- | --- | --- |
| Original.crude | - | - | - |
| Original.crude | Neither frailty nor sarcopenia | Reference |  |
| Original.crude | Sarcopenia only | 2.89 (1.53~5.44) | 0.001 |
| Original.crude | Frailty only | 2.47 (1.32~4.62) | 0.005 |
| Original.crude | Both sarcopenia and frailty | 4.52 (2.48~8.24) | <0.001 |
| Multivariable.adjusted | - | - | - |
| Multivariable.adjusted | Neither frailty nor sarcopenia | Reference |  |
| Multivariable.adjusted | Sarcopenia only | 2.71 (1.41~5.19) | 0.003 |
| Multivariable.adjusted | Frailty only | 1.95 (1.02~3.76) | 0.045 |
| Multivariable.adjusted | Both sarcopenia and frailty | 3.18 (1.66~6.07) | <0.001 |
| Weighted.IPTW | - | - | - |
| Weighted.IPTW | Neither frailty nor sarcopenia | Reference |  |
| Weighted.IPTW | Sarcopenia only | 3.95 (1.8~8.63) | 0.001 |
| Weighted.IPTW | Frailty only | 1.75 (0.89~3.46) | 0.105 |
| Weighted.IPTW | Both sarcopenia and frailty | 3.4 (1.71~6.74) | 0.001 |
| Weighted.overlap | - | - | - |
| Weighted.overlap | Neither frailty nor sarcopenia | Reference |  |
| Weighted.overlap | Sarcopenia only | 2.71 (1.32~5.58) | 0.007 |
| Weighted.overlap | Frailty only | 1.78 (0.87~3.61) | 0.112 |
| Weighted.overlap | Both sarcopenia and frailty | 3.02 (1.52~6.02) | 0.002 |
| Weighted.matching | - | - | - |
| Weighted.matching | Neither frailty nor sarcopenia | Reference |  |
| Weighted.matching | Sarcopenia only | 2.22 (1.06~4.62) | 0.034 |
| Weighted.matching | Frailty only | 1.59 (0.74~3.4) | 0.236 |
| Weighted.matching | Both sarcopenia and frailty | 2.62 (1.26~5.46) | 0.01 |
| Weighted.entropy | - | - | - |
| Weighted.entropy | Neither frailty nor sarcopenia | Reference |  |
| Weighted.entropy | Sarcopenia only | 3.47 (1.6~7.53) | 0.002 |
| Weighted.entropy | Frailty only | 1.82 (0.92~3.62) | 0.088 |
| Weighted.entropy | Both sarcopenia and frailty | 3.23 (1.64~6.36) | 0.001 |
| Weighted.treated | - | - | - |
| Weighted.treated | Neither frailty nor sarcopenia | Reference |  |
| Weighted.treated | Sarcopenia only | 6.91 (2~23.89) | 0.002 |
| Weighted.treated | Frailty only | 2.79 (1.18~6.64) | 0.02 |
| Weighted.treated | Both sarcopenia and frailty | 3.87 (1.77~8.43) | 0.001 |

IPTW, inverse probability treatment weighting.

Supplementary Figure 3. Empirical receiver‑operating‑characteristic (ROC) curves illustrating the predictive performance of unadjusted frailty, sarcopenia, and combined sarcopenia‑frailty scores for worsening disability or death at 3‑month follow‑up after surgery. AUC, area under the curve.

B

A

C

Supplementary Figure 4. (A) Association between preoperative CGA‑FI scores and grip strength (weak correlation). (B) Association between preoperative CGA‑FI scores and skeletal muscle index (weak correlation). (C) Association between preoperative CGA‑FI scores and Gait speed (moderate correlation). Correlation analyses were performed using Spearman’s correlation coefficient.

Supplementary Table 4 Group-based trajectory model fits.

| G | loglik | conv | npm | AIC | BIC | SABIC | entropy |
| --- | --- | --- | --- | --- | --- | --- | --- |
| 2 | -4986.66 | 1 | 15 | 10003.31 | 10067.21 | 10019.59 | 0.903803 |
| 3 | -4828.88 | 1 | 20 | 9697.765 | 9782.957 | 9719.472 | 0.828023 |
| 4 | -4757.17 | 1 | 25 | 9564.335 | 9670.824 | 9591.468 | 0.81249 |

AIC, akaike information criterion; BIC, bayesian information criterion.

Supplementary Table 5 Characteristics of WHODAS trajectory groups.

| Variables | low-stable trajectory (n = 175) | medium-stable trajectory (n = 230) | high-stable trajectory(n = 68) | extremely high-volatile group (n = 50) | P |
| --- | --- | --- | --- | --- | --- |
| Age, Mean ± SD | 65.56 ± 5.16 | 67.08 ± 5.12 | 68.29 ± 5.80 | 68.50 ± 5.72 | <.001 |
| BMI, Mean ± SD | 24.55 ± 3.56 | 24.64 ± 3.35 | 24.34 ± 3.60 | 23.16 ± 3.48 | 0.052 |
| Smoke, n(%) | 72 (41.14) | 76 (33.04) | 16 (23.53) | 14 (28.00) | 0.042 |
| Alcohol, n(%) | 58 (33.14) | 47 (20.43) | 8 (11.76) | 14 (28.00) | 0.001 |
| EuroSCORE II, M (Q₁, Q₃) | 1.09 (0.78,1.40) | 1.25 (0.94,1.71) | 1.62 (1.25,2.11) | 1.83 (1.40,2.62) | <.001 |
| Type of operation, n(%) |  |  |  |  | 0.244 |
| CABG | 100 (57.14) | 139 (60.43) | 35 (51.47) | 19 (38.00) |  |
| Valve | 61 (34.86) | 75 (32.61) | 26 (38.24) | 25 (50.00) |  |
| CABG+Valve | 11 (6.29) | 13 (5.65) | 5 (7.35) | 5 (10.00) |  |
| Other | 3 (1.71) | 3 (1.30) | 2 (2.94) | 1 (2.00) |  |
| Albumin, Mean ± SD | 42.38 ± 3.73 | 41.82 ± 3.45 | 40.72 ± 3.04 | 41.40 ± 3.69 | 0.009 |
| Haemoglobin, Mean ± SD | 138.74 ± 15.08 | 132.86 ± 15.24 | 128.26 ± 16.29 | 129.71 ± 18.16 | <.001 |
| SCR, Mean ± SD | 65.44 ± 16.11 | 64.79 ± 19.00 | 63.54 ± 19.51 | 73.49 ± 30.22 | 0.029 |
| ASA, n(%) |  |  |  |  | <.001 |
| 2 | 8 (4.57) | 8 (3.48) | 0 (0.00) | 1 (2.00) |  |
| 3 | 161 (92.00) | 191 (83.04) | 45 (66.18) | 25 (50.00) |  |
| 4 | 6 (3.43) | 31 (13.48) | 23 (33.82) | 24 (48.00) |  |
| NYHA, n(%) |  |  |  |  | <.001 |
| 2 | 152 (86.86) | 139 (60.43) | 27 (39.71) | 21 (42.00) |  |
| 3 | 23 (13.14) | 90 (39.13) | 39 (57.35) | 26 (52.00) |  |
| 4 | 0 (0.00) | 1 (0.43) | 2 (2.94) | 3 (6.00) |  |
| Sarcopenia frailty score, n(%) |  |  |  |  | <.001 |
| Neither frailty nor sarcopenia | 141 (80.57) | 102 (44.35) | 13 (19.12) | 10 (20.00) |  |
| Sarcopenia only | 5 (2.86) | 52 (22.61) | 18 (26.47) | 5 (10.00) |  |
| Frailty only | 23 (13.14) | 39 (16.96) | 20 (29.41) | 11 (22.00) |  |
| Both sarcopenia and frailty | 6 (3.43) | 37 (16.09) | 17 (25.00) | 24 (48.00) |  |

Data: Means ± standard deviations, absolute rate, and percentage. Statistical significance was defined as p < 0.05. BMI, body mass index; EuroSCORE II, European System for Cardiac Operative Risk Evaluation II; ASA, American Society of Anesthesiologists; NYHA, New York Heart Association; CABG, coronary artery bypass graft.
